# Insights into the deglacial variability of phytoplankton community structure in the eastern equatorial Pacific Ocean using [^231^Pa/^230^Th]xs and opal-carbonate fluxes

**DOI:** 10.1038/s41598-022-26593-1

**Published:** 2022-12-23

**Authors:** Danielle Schimmenti, Franco Marcantonio, Christopher T. Hayes, Jennifer Hertzberg, Matthew Schmidt, John Sarao

**Affiliations:** 1 Department of Geology and Geophysics, Texas A&M University, College Station, TX USA; 2 School of Ocean Science and Engineering, University of Southern Mississippi, Stennis Space Center, MS USA; 3 International Ocean Discovery Program, College Station, TX USA; 4 Department of Ocean and Earth Sciences, Old Dominion University, Norfolk, VA USA

**Keywords:** Climate sciences, Biogeochemistry, Palaeoceanography

## Abstract

Fully and accurately reconstructing changes in oceanic productivity and carbon export and their controls is critical to determining the efficiency of the biological pump and its role in the global carbon cycle through time, particularly in modern CO_2_ source regions like the eastern equatorial Pacific (EEP). Here we present new high-resolution records of sedimentary ^230^Th-normalized opal and nannofossil carbonate fluxes and [^231^Pa/^230^Th]xs ratios from site MV1014-02-17JC in the Panama Basin. We find that, across the last deglaciation, phytoplankton community structure is driven by changing patterns of nutrient (nitrate, iron, and silica) availability which, in turn, are caused by variability in the position of the Intertropical Convergence Zone (ITCZ) and associated changes in biogeochemical cycling and circulation in the Southern Ocean. Our multi-proxy work suggests greater scrutiny is required in the interpretation of common geochemical proxies of productivity and carbon export in the EEP.

## Introduction

The eastern equatorial Pacific (EEP) is a major source of CO_2_ to the atmosphere today^1,2^ due to an inefficient biological pump. Here, primary productivity is limited by the availability of the micronutrient iron^3,4^, despite high rates of upwelling, resulting in low fluxes of organic carbon from surface to depth and net outgassing of carbon dioxide to the atmosphere. Driving the availability of both macro (nitrate and phosphate) and micro (silica and iron) nutrients over the EEP are changes in nutrient utilization over the Southern Ocean^5,6^, equatorial thermocline depth and upwelling rates^7,8^, and windblown dust flux to EEP surface waters^9–11^. The response of the phytoplankton community to these changes can be variable and dynamic with relative availability of N:Si:Fe controlling competition between diatoms and coccolithophores, diatom size, silicification and frustule thickness and, importantly, organic carbon production and ballasting—key to carbon burial and an efficient biological pump^12^. Capturing the dynamics of biological productivity in response to nutrient variability and its drivers is critical to constraining changes in the EEP biological pump and the region’s role in the global carbon cycle in the past, but is often difficult to accomplish.

**Figure 1:**
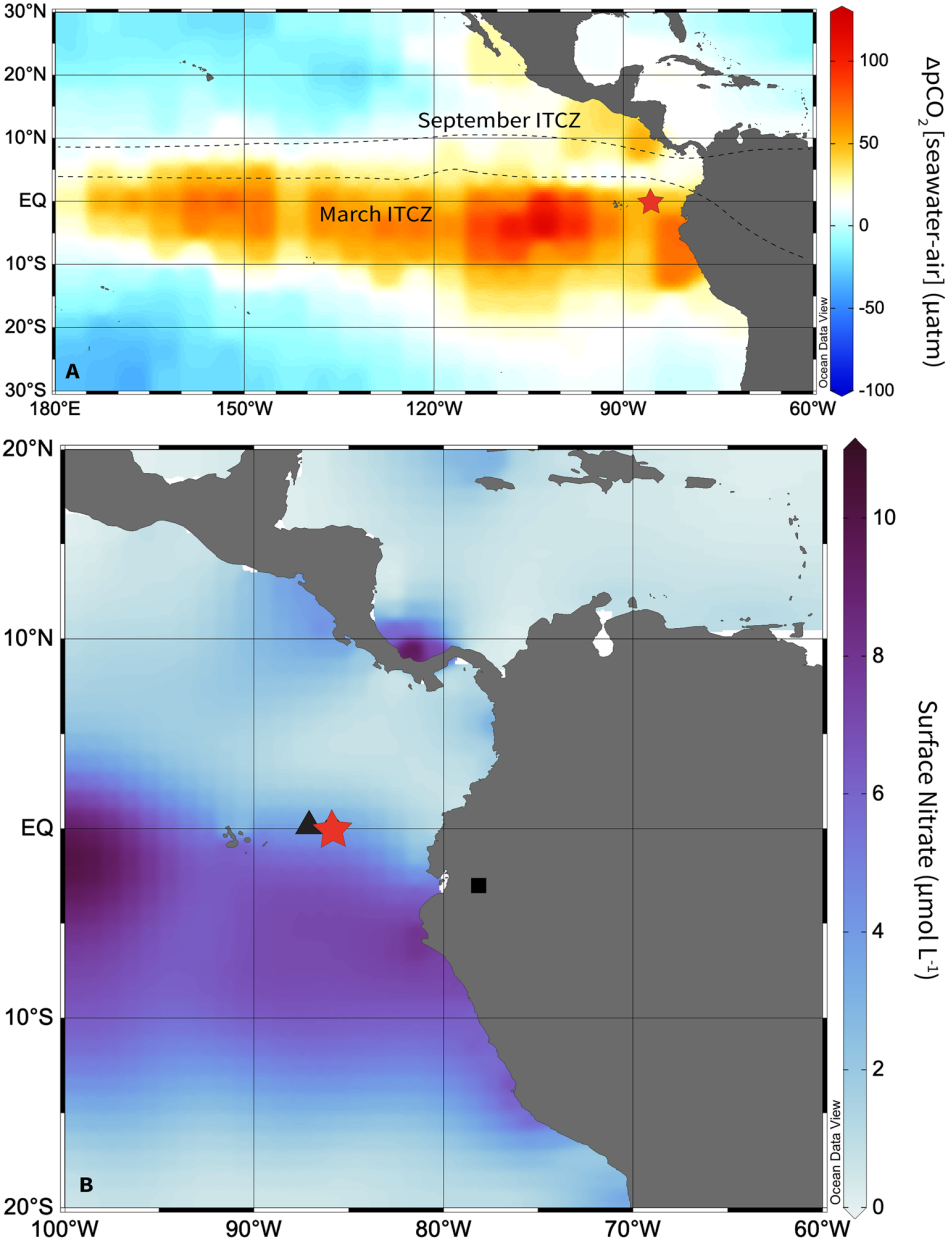
Modern eastern equatorial Pacific hydrography and study site locations. (**A**) ΔpCO_2_ [seawater-air] flux from Takahashi et al.^2^ with location of study site MV1014-02-17JC (red star) and the maximum (September) and minimum (March) mean seasonal positions of the ITCZ (black dashed lines)^34^. (**B**) Mean annual ocean surface nitrate concentrations^69^ with locations of MV1014-02-17JC (red star), ODP 1240 (black triangle)^14,15^, and Santiago Cave (black square)^13^. Maps created in Ocean Data View (Schlitzer, R., version 5.6.2 https://odv.awi.de/).

Here we present new high resolution records of sedimentary [^231^Pa/^230^Th]xs ratios and ^230^Th-normalized biogenic silica (opal) and nannofossil carbonate fluxes from site MV1014-02-17JC (00° 10.8297′ S, 85° 52.0042′ W, 2846 m), south of the Carnegie Ridge in the Panama Basin (Fig. 1), that span the early Holocene to Last Glacial Maximum (LGM), 8–25 ka. We compare our new geochemical and nannofossil proxy records and previously published^11^ MV1014-02-17JC (17JC) records of dust flux (^232^Th flux), export production (xsBa flux), and bottom water oxygenation (authigenic uranium concentrations) to a record of South American Summer Monsoon (SASM) intensity (δ^18^O calcite) from Santiago Cave, Ecuador (3° 1′ S, 78° 8′ W)^13^ and other records of diatom vs. coccolithophore production from adjacent site ODP 1240 (0° 1.31′ N, 86° 27.76′ W; 2921 m) in the Panama Basin^14,15^.

Via this multi-proxy approach, we can reconstruct a more complete picture of the atmosphere–ocean dynamics that governed EEP nutrient availability and consequently phytoplankton community structure and export production across the LGM, deglaciation, and early Holocene. We find that a significant shift in phytoplankton community structure of the EEP occurred over the deglaciation, consistent with previous findings from the region^14,15^; and that nutrient dynamics, driven by variability in the average position of the ITCZ over the EEP and climate over the Southern Ocean, were important determinants of phytoplankton community structure and consequently export production in the EEP over the past 25,000 years.

## Results

**Figure 2:**
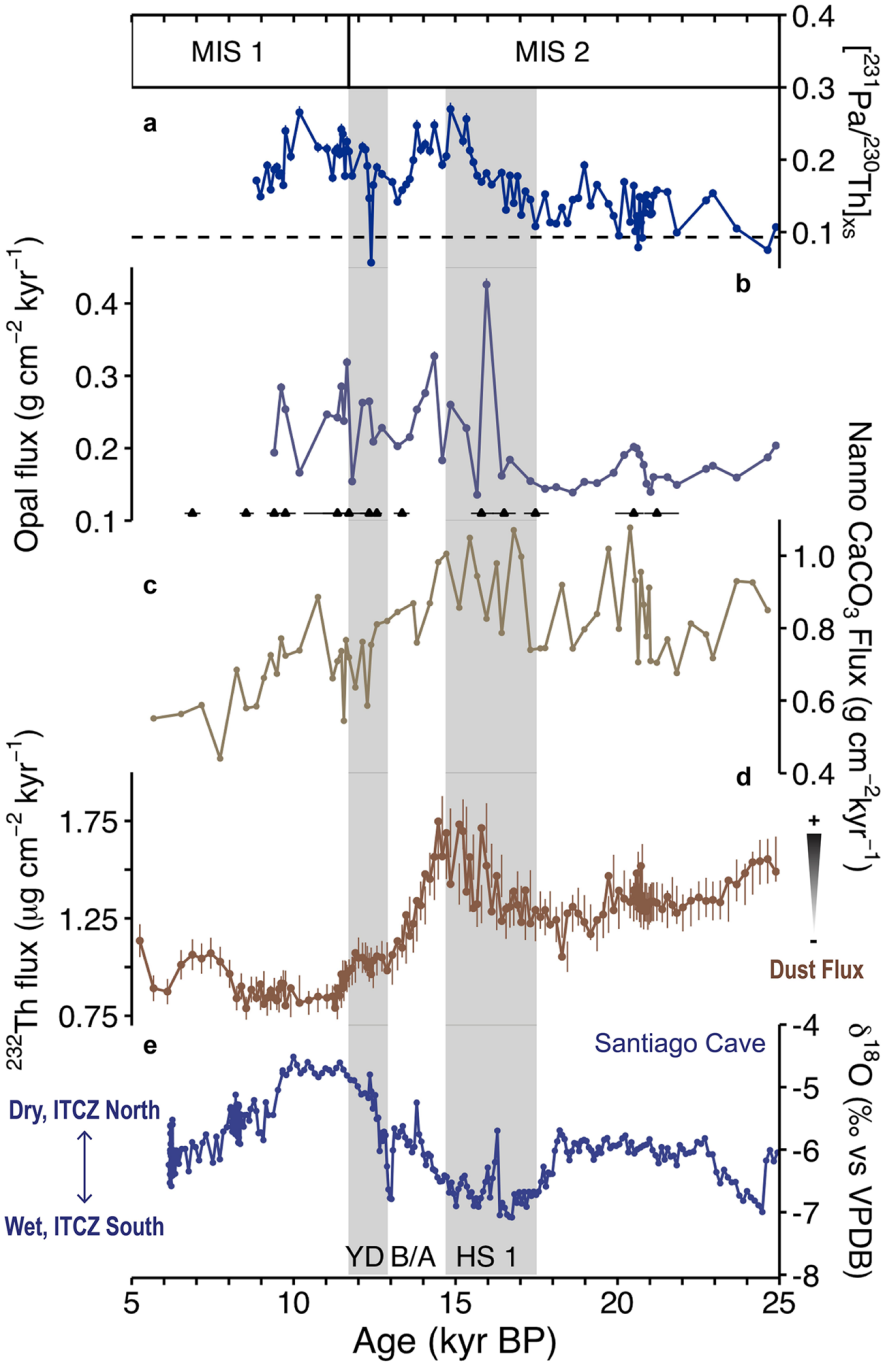
Geochemical proxy records for marine sediment core 17JC and Santiago Cave speleothems shown with Marine Isotope Stage (MIS) boundaries from Lisiecki and Raymo^70^ and abrupt climate events-Heinrich Stadial 1 (HS1), the Bølling-Allerød (B/A), and the Younger Dryas (YD)- of the deglacial from McManus et al.^29^ (grey shaded bars represent cold stadials). (**a**) 17JC Initial [^231^Pa/^230^Th]xs and the ^231^Pa/^230^Th production ratio of 0.093 (dashed black line), (**b**) 17JC ^230^Th-normalized opal fluxes, (**c**) 17JC ^230^Th-normalized nannofossil carbonate fluxes, (**d**) 17JC ^230^Th-normalized ^232^Th (dust) fluxes^11^, and (**e**) Santiago Cave δ^18^O calcite^13^. Vertical lines through data points represent error bars (see Supplemental Table 2); those not visible are smaller than the data point symbol. Black triangles in panel b mark the radiocarbon ages and associated uncertainties (horizontal bars) for 17JC (see “Methods”).

[^231^Pa/^230^Th]xs ratios are above the production ratio (0.093) for the vast majority of the past 25 kyr at 17JC (Fig. 2a), indicating generally high particle fluxes and excess scavenging of ^231^Pa from the water column. We consider initial [^231^Pa/^230^Th]xs a secondary proxy of opal flux^16^ to 17JC sediments (see Supplementary Fig. 2) that is insensitive to remineralization and can be used to corroborate interpretations of ^230^Th-normalized opal fluxes. Sedimentary opal fluxes and nannofossil carbonate fluxes serve as independent records of biological productivity driven by diatom vs. coccolithophore growth in the surface. Nannofossil carbonate fluxes are estimated from the proportion of carbonate in the fine (< 63 µm) fraction of the sediments at 17JC (see “Methods”). Our approach to reconstructing nannofossil carbonate fluxes in this way is somewhat unconventional given the potential caveat of differential dissolution on the preservation of both foraminiferal and coccolith calcite within ocean sediments^17^. However, we are confident that due to the unique locality and sedimentary environment of our site that carbonate dissolution rates were low and unlikely to have affected our record, which is corroborated by evidence of excellent preservation of foraminiferal tests in 17JC sediments (see Supplementary Sect. 1). Grain-size analyses have also shown that the majority (> 90%) of the < 63 µm fraction of the sediment is found within the < 20 µm fraction^18^, such that our estimates are largely representative of the < 20 µm fraction (see “Methods”). Further, the lack of material in the 20–63 µm range where we would expect most foram fragments to lie, if present, leads us to conclude it is unlikely foram fragments constitute a significant portion of the < 63 µm or < 20 µm fraction of the sediments.

Both [^231^Pa/^230^Th]xs and ^230^Th-normalized opal fluxes exhibit similar trends across the last 25 kyr (Fig. 2a,b). Relative to the Holocene, late glacial (~ 22–25 ka) to LGM (~ 21 ka) values are low with [^231^Pa/^230^Th]xs between 0.075 (below the production ratio) and 0.17, and opal fluxes below 0.2 g cm^−2^ kyr^−1^ at the onset of the deglaciation (~ 19–20 ka). Following the onset of the deglaciation, [^231^Pa/^230^Th]xs exhibits two broad peaks at the end of Heinrich Stadial 1 (HS1, ~ 14.7–17.5 ka) and during the early Holocene (~ 10 ka) while opal fluxes undergo an abrupt increase at ~ 16 ka and remain elevated on average (~ 0.25 g cm^−2^ kyr^−1^) through the end of the early Holocene (~ 9 ka). Nannofossil carbonate fluxes at 17JC exhibit an opposite trend across the LGM to early Holocene (Fig. 2c). Nannofossil carbonate fluxes are elevated (~ 1 g cm^−2^ kyr) between ~ 20–14 ka and subsequently undergo a gradual decrease post-HS1 through the remainder of the deglacial to values on average around 0.69 g cm^−2^ kyr.

We also calculate the ratio of opal fluxes to the sum of the opal and nannofossil carbonate fluxes (Fig. 4a) (see “Methods”) to assess the timing of phytoplankton community structure shift. Opal: (Opal + Nannofossil Carbonate) ratios exhibit similar trends as the opal fluxes and [^231^Pa/^230^Th]xs ratios over the past 25,000 years at 17JC as well as that of existing records of diatom to coccolithophore production in the EEP (Fig. 4b).

## Discussion

### The last glacial maximum (LGM)

Low [^231^Pa/^230^Th]xs values at the LGM (Fig. 2a) suggests reduced opal fluxes associated with overall reduced surface productivity, as has previously been suggested for this period over the equatorial Pacific^9,10,19–23^. However, while opal fluxes were low (Fig. 2b), nannofossil carbonate fluxes were elevated at this time (Fig. 2c). This disparity in composition of the biogenic fraction of the sediments suggests a divergence in the phytoplankton community at the LGM, specifically, the dominance of calcareous coccolithophores over siliceous diatoms, as has been identified elsewhere in the EEP during this event^14,15^.

The EEP primarily derives its surface water inventory of nitrate and silicate from the equatorial undercurrent (EUC) that bathes the EEP thermocline from which waters are drawn via Ekman pumping to the surface^6,24^. The EUC, in turn, derives its nutrient inventory from intermediate waters, particularly Subantarctic Mode Waters (SAMW) formed in the subantarctic zone of the Southern Ocean^5^. It has been suggested that Fe fertilization via enhanced dust deposition to waters south of the polar front would have led to increased nitrate utilization relative to silicate by biological producers in the surface, such that SAMW would have formed with high amounts of preformed silicate and low amounts of preformed nitrate at the LGM^5^. Consequently, several studies have concluded that the lack of opal burial at the LGM over the EEP, despite coeval elevated fluxes of dust^9–11^, can be attributed to reduced silica utilization by diatoms under Fe replete conditions—similar to the mechanism acting over diatom productivity in the Southern Ocean at this time--rather than silica limitation^9,15^. However, δ^13^C of thermocline dwelling foraminifera from the EEP have indicated the possibility of reduced deep-water ventilation (and upwelling of nutrients) in the Southern Ocean that led to the transport of SAMW low in nitrate and silica to the EEP at the LGM^14^, one factor that points towards the possibility of silica limitation for EEP diatoms.

Nearby on the South American continent, precipitation records from Santiago Cave indicate a reduction in SASM strength at the LGM^13^ (Fig. 2e), consistent with evidence for a southward displacement of the ITCZ over the South American continent^25^ and the equatorial Pacific^26,27^. The effect of this displacement in the EEP, specifically, was a contraction of the EEP cold tongue caused by reduced cross-equatorial flow of the southern trade winds and a deeper thermocline^7^, which reduced upwelling and surface cooling at the equator.^26^ Not only were upwelled waters possibly low in nutrients (due to decreased ventilation at their source), equatorial upwelling itself was reduced, producing EEP surface waters likely low in silica and nitrate. The inventory of surface water nitrate may have been bolstered by input from the margins at this time^15^, but most importantly LGM silica availability was likely reduced while iron inputs from dust were high. What may have previously been interpreted as reduced silica utilization by diatoms in surface waters resulting in low opal fluxes to EEP sediments at the LGM, may therefore actually be explained by silica limitation.

Low silica coupled with equal or higher relative availability of nitrate in surface waters can explain the divergence in sedimentary inventory of nannofossil carbonate relative to opal we observe at 17JC at the LGM (Fig. 2b,c). Coccolithophores will outcompete diatoms under high iron and low Si:N or equal Si:N conditions^28^, which should lead to high fluxes of nannofossil carbonate and low fluxes of opal to the sediments, whereas iron and silica replete conditions should generally act to promote the growth of large well-silicified diatoms that would outcompete coccolithophores and instead generate high sedimentary opal and low nannofossil carbonate fluxes. We infer the former case of nutrient availability in EEP surface waters at the LGM based on observed sedimentary fluxes at 17JC and elsewhere in the EEP.

### Heinrich Stadial 1 (HS1)

HS1 was characterized by a weakened Atlantic Meridional Overturning Circulation (AMOC)^29^ that triggered Northern Hemisphere (NH) cooling^30^. A wide range of proxy reconstructions and modeling experiments agree that this change in ocean circulation and associated heat transport generated a southward shift of the ITCZ and intensification of the annual-mean Hadley cell in the NH while weakening that of the Southern Hemisphere (SH)^31–33^, which is consistent with evidence for a weakening of NH monsoons^34–36^ and strengthening of the SASM^13,25,37^ (Fig. 2e). Recent studies indicate a weaker (or possibly shutoff) AMOC at HS1^30^ forced the ITCZ further south of its average position during the LGM^38^ and generated comparatively stronger NH but weaker SH trade winds^39^. Cooler SSTs associated with elevated productivity in the EEP cold tongue^23^ support the possibility of enhanced equatorial upwelling and therefore nutrient delivery driven by strengthened NH trade winds at HS1.

In terms of Southern Ocean controls on nutrient delivery to the EEP at HS1, there is evidence that export of intermediate waters to low latitudes was enhanced^40^ but the state of their nutrient content at this time is less clear. While some studies suggest that the silica inventory of SAMW^14,22^ was enhanced over the deglacial, others suggest that a loss of Fe fertilization over the Southern Ocean should have reduced the silica inventory and increased the nitrate inventory of SAMW over the same time period^41^.

Our proxy evidence is indicative of enhanced silica availability but high N:Si ratios in EEP surface waters beginning at HS1. While opal fluxes (and Pa/Th ratios) begin to rise (Fig. 2a,b), nannofossil carbonate fluxes remain elevated on average, similar to their LGM values (Fig. 2c), suggesting the continued dominance of coccolithophores in the surface despite apparent increases in diatom production. Dust fluxes also increase to peak values at this time (Fig. 2d), indicating enhanced Fe delivery to EEP surface waters relative to the LGM, such that iron likely remained a non-limiting nutrient for primary production at HS1.

If nutrient concentrations of Southern Ocean surface waters were, in fact, significantly enhanced due to reinvigoration of deep-water ventilation at the onset of the deglaciation, SAMW may have formed with higher silica concentrations relative to the LGM despite potential increases in the N:Si ratio fostered by changes in the relative utilization of nitrate vs. silica south of the polar front. Consequently, this increase in silica concentration of waters reaching the EEP thermocline together with enhanced equatorial upwelling caused by a southward shift of the ITCZ may have been sufficient to stimulate increases in diatom production and opal flux to EEP sediments while the high N:Si ratio of upwelled waters prevented diatoms from fully outcompeting coccolithophores in the surface at HS1. This is consistent with evidence from nearby site ODP 1240 that diatom silicification increased in the EEP across HS1^15^. Still, more evidence is needed from the Southern Ocean to corroborate our hypothesis on nutrient inventory variability of SAMW across the deglaciation.

**Figure 3:**
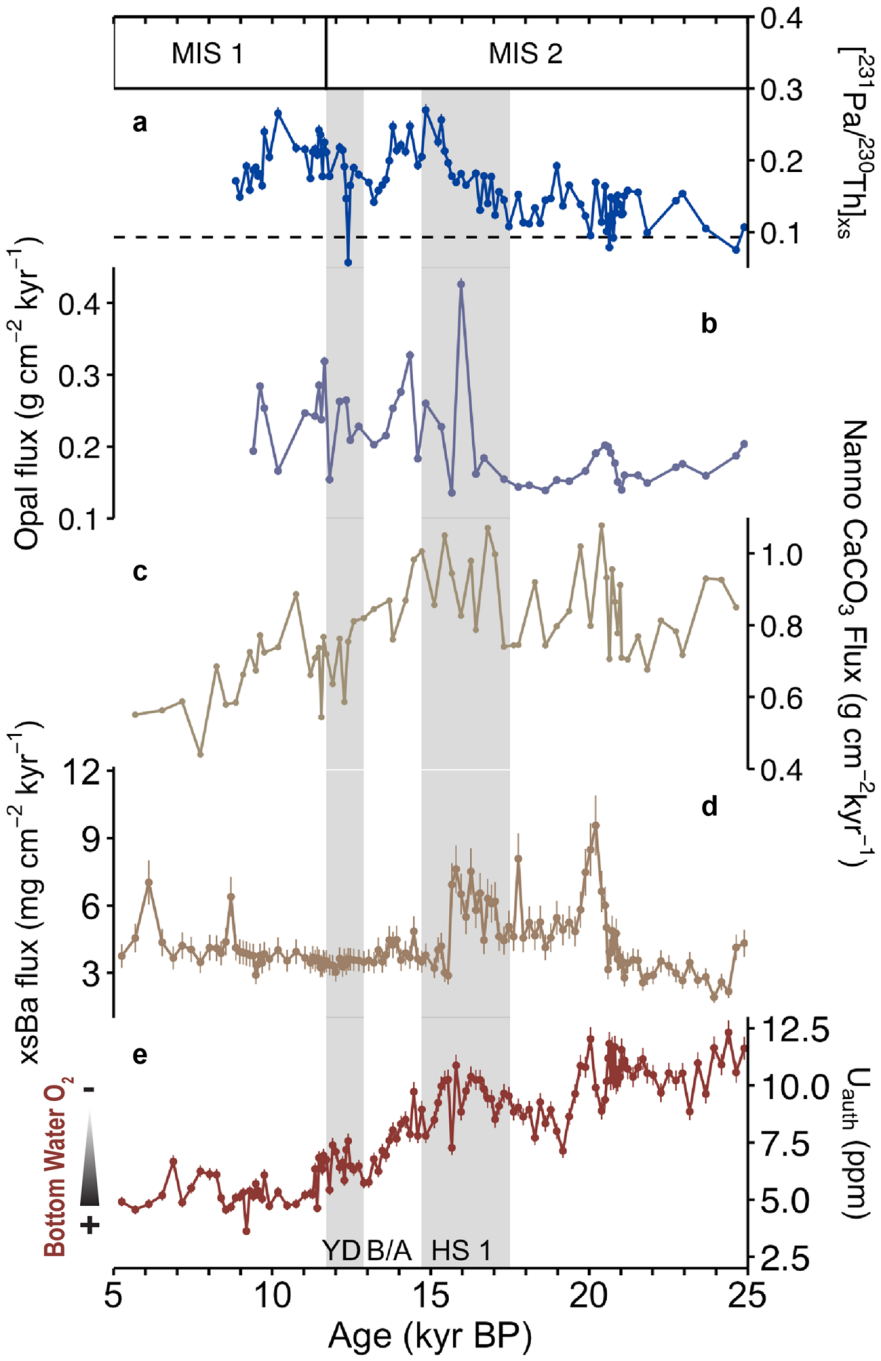
Proxy records of export production and bottom water oxygenation state at 17JC shown with MIS boundaries from Lisiecki and Raymo^70^ and HS1, B/A, and YD boundaries from McManus et al.^29^ (grey shaded bars represent cold stadials). (**a**) Initial [^231^Pa/^230^Th]xs and the ^231^Pa/^230^Th production ratio of 0.093 (dashed black line), (**b**) ^230^Th-normalized opal fluxes, (**c**) ^230^Th-normalized nannofossil carbonate fluxes, (**d**) ^230^Th-normalized xsBa (organic matter) fluxes^11^, and (**e**) Authigenic uranium (Uauth) concentrations^11^, a proxy for bottom water oxygenation. Lines through data points represent error bars (see methods); those not visible are smaller than the data symbol.

Along with this change in nutrient availability, there appears to have been a threshold on organic matter flux that led to diagenetic loss of barite in the sediment column during HS1 at 17JC. Despite elevated productivity (Fig. 3a–c) and, presumably, flux of organic matter that would generally act to deplete oxygen and therefore elevate authigenic uranium (U_auth_) with the reduction of U(VI) to U(IV) in the sediments, U_auth_ concentrations steadily decline with re-ventilation of deep waters at the onset of the deglaciation^11,42^ (Fig. 3e). This trend should have continued through the remainder of the deglaciation, but instead U_auth_ increases during HS1 when opal fluxes double (Fig. 3b). Lacking any direct evidence of redox state of the sediments, it appears that organic matter fluxes are high enough at this time to drive redox changes in the sediment column.

Crystalline barite (BaSO_4_) precipitates within saturated microenvironments of settling particles in the water column by the interaction of Ba^2+^ present in seawater with SO_4_^2−^ supplied by decaying organic matter^43,44^ such that its flux to sediments is considered a good proxy for organic carbon fluxes through time^45,46^. xsBa fluxes at 17JC remain elevated through HS1 until ~ 15 ka when the flux of barite abruptly decreases and remains low thereafter (Fig. 3d). Barite is generally considered refractory in sediments, but given high enough sedimentation rates, the sediment column can become so depleted of oxygen that BaSO_4_ will be consumed with sulfate reduction^47^. We therefore assume high production rates of barite in the water column above 17JC with high organic matter fluxes^48^ (driven by the addition of enhanced opal production) through the end of HS1 and onset of the B/A (~ 14 ka), but that the barium signal in the sediments is most likely lost with redox driven consumption of barite in the sediment column during peak barite/ organic matter fluxes ~ 15 ka, as suggested by Marcantonio et al.^42,49^.

### Post-HS1 and the early holocene

The relationship between ITCZ position, upwelling, and productivity in the EEP is somewhat less clear over the remainder of the deglacial. Precipitation records from the Cariaco basin^50^ and Santiago Cave^13^ (Fig. 2e) suggest that abrupt warming of the northern hemisphere during the Bølling-Allerød (B/A, ~ 14.7 ka) may have resulted in a northward migration of the ITCZ from its position at HS1. A northward shift of the ITCZ would be expected to reverse the effect on the trade winds^51^ during HS1, weakening the NH trades and strengthening the SH trades. However, available SH trade wind proxy records post-HS1 are not coherent in terms of fluctuations in trade wind strength during interstadials^39^. Therefore, it is unclear how equatorial upwelling varied across this timeframe. Still, dust fluxes, opal fluxes, nannofossil carbonate fluxes, and [^231^Pa/^230^Th]xs all decline at 17JC (Fig. 2a, b, c, d) at the onset of the B/A. The decline in dust flux is likely driven by a change in dust source from more NH sources at HS1 to more southern hemisphere sources at B/A with the northward migration of the ITCZ (a barrier to cross-equatorial wind flow); however, given a lack of constraints on the magnitude of the ITCZ shift between HS1 and the B/A, we cannot rule out a change in trade wind strength as a control on dust fluxes to our site. Nonetheless, loss of Fe flux to EEP surface waters from dust is not sufficient to explain low opal fluxes and nannofossil carbonate fluxes during the B/A. Changes in nitrate/silicate delivery would also be necessary. SAMW export to the EEP is reduced during interstadials^40^, which could explain the change in delivery of these key nutrients, barring any changes in upwelling.

Despite evidence from the tropical Atlantic for another abrupt southward shift of the ITCZ at the Younger Dryas (YD, ~ 12.9–11.7 ka)^50^, Santiago Cave precipitation indicates continued northward migration of the ITCZ across the YD to peak northern displacement at the early Holocene (~ 10 ka) (Fig. 2e). Similarly, biogenic and terrigenous fluxes to 17JC sediments are not indicative of an abrupt change associated with ITCZ migration at the YD. While 17JC dust fluxes and nannofossil carbonate fluxes remain low (Fig. 2c,d), seemingly unimpacted by abrupt NH cooling at the YD, opal fluxes and [^231^Pa/^230^Th]xs ratios begin to rise (Fig. 2a,b), reaching peaks in the early Holocene comparable to those observed at HS1, which we interpret as evidence for a switch to diatom dominance in the phytoplankton community at the early Holocene as has been identified at other sites in the EEP^14,15^. Given a lack of iron delivery by dust at this time, the rise in opal must be explained by enhanced diatom production driven by increased silica delivery to EEP surface waters.

Northern displacement of the seasonal extent of the ITCZ at the early Holocene can be explained by warming of the northern extratropics relative to the southern extratropics with perihelion occurring during boreal summer at ~ 10 ka^52^. Lacking any direct proxy evidence of trade wind strength for this period, we might assume a strengthening of the SH trade winds associated with summertime cooling of the SH relative to the NH and significant northward displacement of the ITCZ such that cross-equatorial flow of the SH trades enhanced upwelling at the equator. Further, SAMW export to the EEP was highest at 10 ka^40^, and therefore could have enhanced silica delivery to EEP surface waters.

What is lacking in 17JC sediments at the early Holocene is a coeval peak in xsBa associated with the peak in opal flux and [^231^Pa/^230^Th]xs ratios. Presumably, if diatom production was enhanced, it should have driven an increase in organic matter flux and enhanced barite production in the water column (and its flux to the sediments). However, this is not what we observe. Given no evidence for oxygenation changes that might lead to post-depositional consumption of barite in the sediment column (Fig. 3e), we assume organic matter production in the surface was too low to support significant production of barite in the water column at the early Holocene. This hypothesis is supported by evidence for regionally low Holocene organic carbon fluxes across the EEP^15^.

Why were organic carbon fluxes low when diatom production was elevated at the early Holocene? For one, this could be caused by the composition of the diatom community itself. High silica but low iron availability, such as what likely existed in EEP surface waters due to enhanced upwelling but decreased dust fluxes at the early Holocene, may have supported the growth of large heavily silicified diatoms with high Si:C content. These “Si-sinkers” are resistant to grazing by large zooplankton and sink efficiently due to their size such that their growth is associated with high opal but comparatively low organic carbon export out of the surface^12^. This coupled with an apparent lack of coccolithophore production (and its associated contribution to organic carbon export) at the early Holocene may explain low organic carbon and xsBa fluxes found in EEP sediments. Consequently, we would conclude that the early Holocene peaks in opal flux and [^231^Pa/^230^Th]xs are not indicative of enhanced export production and carbon burial, but rather enhanced diatom production driven by increased upwelling and silica delivery to surface waters.

This is an important finding as opal fluxes and xsBa fluxes have often been employed as independent proxies of export production, particularly within the EEP. We have shown that there are times when relative variations in opal and xsBa fluxes at the same site do not agree often due to effects unrelated to variability in export production. In general, one must be careful when using the xsBa as a proxy for export production across the deglaciation in the EEP, particularly within the Panama Basin where periodically low bottom water oxygenation and extremely high productivity compromise its preservation in the sediments^42,49^. Further, a similar caution should be applied when using sedimentary opal fluxes alone (even when paired with [^231^Pa/^230^Th]xs ratios) to make interpretations about overall export production at sites throughout the global ocean, but particularly within the tropical Pacific where variability in nutrient availability appears to not only drive changes in phytoplankton community composition but also decouple silica export from organic carbon export at times.

### Long-term drivers of EEP productivity

Precessional insolation forcing on the mean latitudinal position of the ITCZ has been invoked as a key driver of both tropical hydroclimate on the South American continent^13,53,54^ and Pacific upper ocean dynamics^55^ over the Pleistocene, and it has been identified as a potential mode of productivity variability over the past 30 kyr in the EEP^20^. As discussed, latitudinal migrations of the ITCZ act as an important control on upwelling intensity at the equator and silica delivery that principally limits diatom productivity and opal flux. Therefore, we might expect that variability in EEP productivity is also sensitive to changes in insolation affecting ITCZ position.

**Figure 4:**
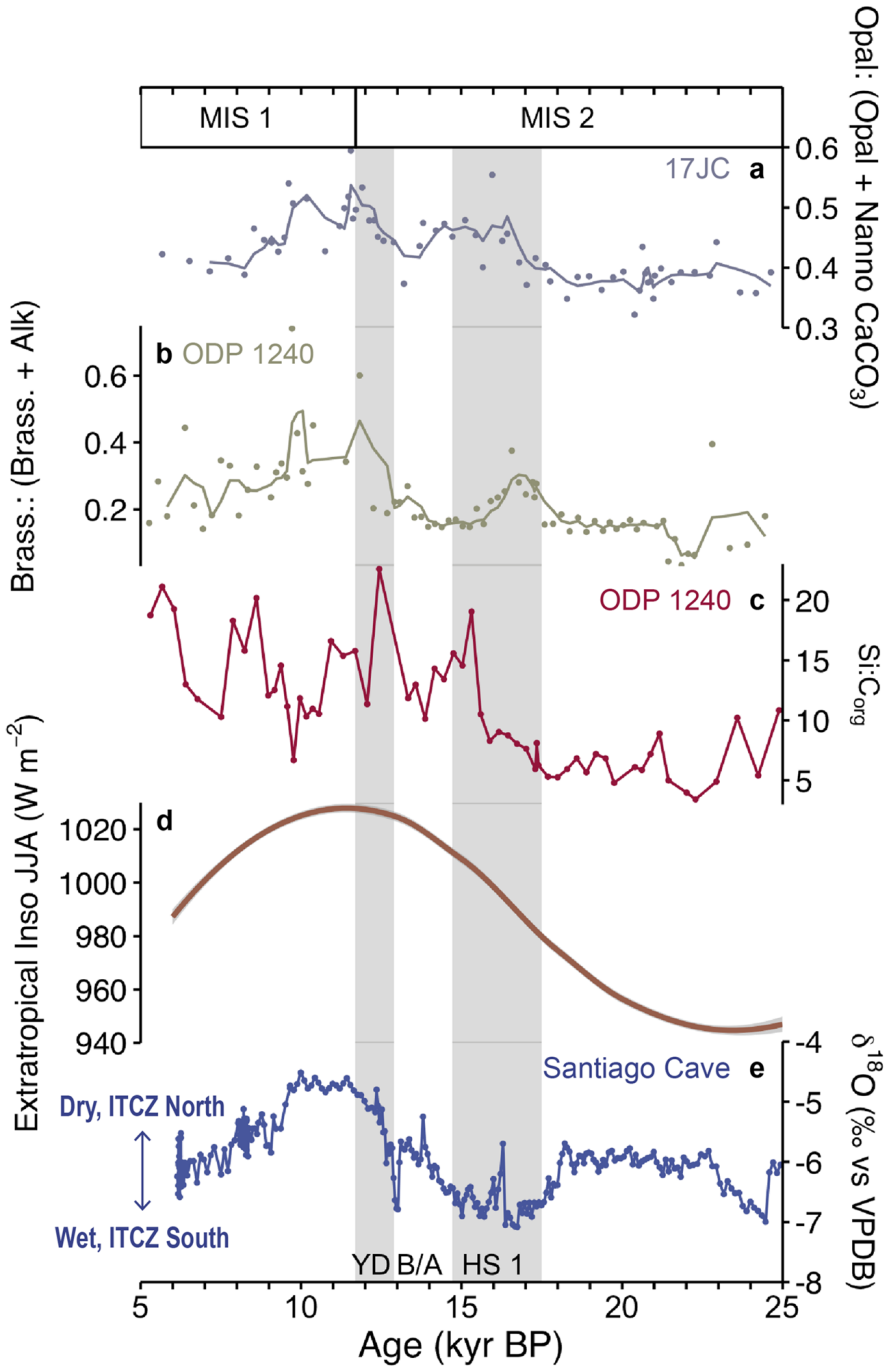
Comparison of phytoplankton community structure shift in the EEP with variability in boreal summer extratropical insolation contrast and ITCZ position over the past 25 kyr. (**a**) 17JC Opal: (Opal + Nannofossil Carbonate) ratios (see “Methods”). The line through the data points represents a three-point running mean. (**b**) ODP 1240 brassicasterol: (brassicasterol + alkenone) ratios^14^. The line through the data points represents a three-point running mean. (**c**) ODP 1240 sedimentary silica: organic carbon (Si:C_org_) ratios^15^. (**d**) Difference in mean boreal summer insolation between 30° N and 30° S generated in QAnalyseries^71^ based on solutions from Laskar et al.^72^ and plotted as the local polynomial regression fitting (loess) of the data with grey 2-sigma error window. (**e**) Santiago Cave δ^18^O calcite^13^. MIS boundaries are from Lisiecki and Raymo,^70^ and HS1, B/A, and YD boundaries are from McManus et al.^29^.

We calculate Opal: (Opal + Nannofossil Carbonate) ratios to more broadly assess the timing of the deglacial shift in phytoplankton community structure and its possible drivers. The LGM to early Holocene trend in Opal: (Opal + Nannofossil Carbonate) ratios at 17JC is, in fact, remarkably consistent with variability in biomarker reconstructions of diatom:coccolithophore production [brassicasterol: (brassicasterol + alkenones)] at adjacent site ODP 1240 and the boreal summer extratropical insolation contrast (North minus South) over the past 25,000 years (Fig. 4a,b,d). We consider this insolation record, in particular, due to the well-established influence of interhemispheric temperature gradients, and energy export out of the extratropics (poleward of 24° N and 24° S), on determining the cross-atmospheric energy flux and therefore ITCZ position^52^. 17JC Opal: (Opal + Nannofossil Carbonate) ratios and ODP 1240 brassicasterol: (brassicasterol + alkenones) ratios reach minimum values (Fig. 4a,b) with minimum extratropical insolation contrast (Fig. 4d) and presumably northward cross-equatorial energy flux that drives the ITCZ south (Fig. 4e) towards the warmer Southern Hemisphere at the LGM. Maximum 17JC Opal: (Opal + Nannofossil Carbonate) and ODP 1240 brassicasterol: (brassicasterol + alkenones) values occur during peak insolation contrast (Fig. 4a,b,d) and enhanced southward cross-equatorial energy flux (relative to today)^56^ that drives the ITCZ further north (Fig. 4e) of its modern day position in the warmer Northern Hemisphere^52^ in the early Holocene.

Overall, the co-variations we observe between phytoplankton community structure and extratropical insolation are consistent with a deglacial transition from an ITCZ, on average, further south at the LGM that causes decreased equatorial upwelling and diatom production to an ITCZ, on average, further north in the early Holocene that leads to high upwelling and enhanced diatom production. The onset of HS1 marks the transition in upwelling and silica delivery between the Holocene and LGM that sparks the first pulse of enhanced diatom production we observe in the early deglacial (~ 18–14 ka), but the shift in phytoplankton community structure from a coccolithophore- to a diatom-dominant community is not fully realized until the onset of the Holocene when high upwelling coupled with low dust fluxes create the perfect recipe for this change. This timing is consistent with the transition from low to high silica: organic carbon (Si:C_org_) ratios in EEP sediments occurring during HS1 (Fig. 4c) but peaks in opal: (opal + nannofossil carbonate) and brassicasterol: (brassicasterol + alkenones) centered at the early Holocene (Fig. 4a,b).

Unfortunately, our record is too short (less than a full precessional cycle) to confirm the 23-kyr precessional mode of variability in phytoplankton community structure for the EEP, but orbital forcing certainly merits further consideration in equatorial Pacific records of biogenic fluxes that cover multiple glacial-interglacial cycles of the Pleistocene.

Our results reveal that deciphering the vagaries of EEP productivity over the deglaciation is not necessarily straightforward, and that the contributions of calcareous and siliceous producers, along with concomitant nutrient dynamics, must be considered to fully capture the complexity of the phytoplankton community ultimately driving variations in export production. This advocates strongly, therefore, for a multi-proxy approach to reconstructing past changes in productivity, particularly in the EEP. Further, our approach (using sedimentary [^231^Pa/^230^Th]xs ratios, opal fluxes, and nannofossil (fine fraction) carbonate fluxes) to reconstruct phytoplankton community shifts may be uniquely applicable to this corner of the EEP. However, we believe this approach is worth considering for future paleoceanographic studies in other localities where biogenic silica, carbonate, and Pa and Th isotope data are readily available; so long as the potential for dissolution is carefully considered and shown to have little influence such as at our site.

## Methods

### Site and sampling

Deep-sea sediment core MV1014-02-17JC was retrieved from south of the Carnegie Ridge in the Panama Basin (00° 10.8297′ S, 85° 52.0042′ W) and a water depth of 2846 m (Fig. 1). The average sedimentation rate at site 17JC is 18 cm kyr^−1^, corresponding to an age resolution of ~ 60–500 year for an average sampling resolution of 3 cm downcore.

### Radiocarbon ages and age model

Eleven new radiocarbon ages were obtained for core 17JC from analyses of the planktic foraminifer *Neogloboquadrina dutertrei* (> 250 µm) at the NOSAMS Woods Hole Oceanographic Institution. *N. dutertrei* were picked from sediment samples spanning the deglacial (12–19 ka) to the Holocene (0–12 ka) (age constraints from Marcantonio et al.^42^). The new radiocarbon ages together with existing radiocarbon ages^11^ obtained for the deglacial to LGM (19–21 ka) were calibrated to calendar year using the Marine20 calibration curve and its built-in 500-year reservoir age correction. The new age model for the Holocene to LGM was constructed using the Bayesian statistical package for age depth modelling ‘rbacon’^57^ in R with a section thickness of 10 cm and an average accumulation rate of 50 year cm^−1^ (Supplemental Data Table 3). 4000 Markov Chain Monte Carlo (MCMC) iterations yielded a Gelman and Rubin Reduction Factor of 1.038, below the 1.05 safety threshold, indicating robust MCMC mixing for our model. Calibrated age errors were calculated by the model based on a 95% confidence interval, which yielded an average uncertainty for the radiocarbon ages of + 320 years/− 397 years and + 574 years/− 602 years for all of the ages determined by the model (see Supplemental Data Table 2).

### [^231^Pa/^230^Th]xs ratios

Pa chemistry was performed between the Hayes Lab for Marine Geochemistry at the University of Southern Mississippi and the Williams Radiogenic Isotope Laboratory at Texas A&M University. Bulk, homogenized sediment was spiked and equilibrated with ^233^Pa and brought to total dissolution using a combination of HNO_3_, HF, HCl, and either H_2_O_2_ or HClO_4_. ^231^Pa and ^233^Pa were separated from other U-series species by ion-exchange chromatography following Fe co-precipitation and subsequently analyzed on the magnetic sector ELEMENT XR Inductively Coupled Plasma Mass Spectrometer (ICP-MS) in the Williams Radiogenic Isotope Laboratory at Texas A&M University. Downcore measurements of ^238^U and ^232^Th for 17JC from Loveley et al.^11^ were used to estimate supported, detrital, and ingrown ^231^Pa and ^230^Th activities and to calculate initial [^231^Pa/^230^Th]xs ratios. Twenty-five replicate analyses of ^231^Pa were used to calculate the average reproducibility of the [^231^Pa/^230^Th]xs ratio for the entire core record, discarding four replicate values that fell outside the two sigma error. In so doing, we calculate a relative standard error of the [^231^Pa/^230^Th]xs replicate analyses of ± 3.2%.

We calculated average [^231^Pa/^230^Th]xs ratios for the past 25 kyr at 17JC and compared these to values at other sites within the EEP (Supplemental Fig. 2). The strongest correlation over the past 25 kyr across the EEP between fluxes of the various sedimentary fractions and [^231^Pa/^230^Th]xs ratios exists between opal fluxes and [^231^Pa/^230^Th]xs with data from 17JC fitting well within the regression, indicating [^231^Pa/^230^Th]xs at 17JC is a good secondary proxy for opal flux and is not sensitive to changes in bulk fluxes (MARs) or particle composition over this timeframe.

### Opal fluxes

Bulk sedimentary opal concentration analyses were performed at the International Ocean Discovery Program Gulf Coast Repository using an Agilent Technologies Cary 100 double beam UV–Vis spectrophotometer. Opal concentrations were calculated as % biogenic opal (dry weight) measured by alkaline extraction and molybdate-blue spectrophotometry following Mortlock and Froelich^58^. ^230^Th-normalized opal fluxes were calculated by multiplying % biogenic opal by the downcore ^230^Th-normalized mass accumulation rates (MARs) for 17JC from Loveley et al.^11^. Nine replicate analyses of % opal were used to calculate average reproducibility for the ^230^Th-normalized opal fluxes spanning the entire core record, discarding three values that fell outside the two sigma error. The relative standard error from replicates was ± 2.02%.

Following the work of Thiagarajan and McManus^59^ in the EEP, expected opal fluxes were estimated by assuming a sediment composition of biogenic carbonate, opal, and lithogenic dust (< 5 µm), such that all fractions summed to 1 per the following equation:

$${\text{Expected}}\,\% \,{\text{opal }} = { 1 }{-} \, \left( {{\text{wt}}\% \,{\text{CaCO}}_{{3}} + \, \% \,{\text{detrital}}} \right),$$

and we estimate % detrital values for each sample by:

$$\% \,{\text{detrital }} = {\text{ measured}}^{{{232}}} {\text{Th}}\,{\text{activity }}\left( {{\text{dpm}}\,{\text{g}}^{{ - {1}}} } \right) \, \times { 4}.{1}0{661 } \times { 14}\,{\text{ppm}},$$

where 14 ppm is the concentration of ^232^Th of fine grained dust per McGee et al.^60^. Good agreement between the relative variations in the expected and measured opal fluxes over the past 25,000 years at 17JC (Supplemental Fig. 3) allowed us to utilize expected opal fluxes to fill in gaps in our measured opal flux data when calculating opal: (opal + nannofossil carbonate) ratios (Fig. 4a).

### Carbonate fluxes

Bulk sedimentary % calcium carbonate (CaCO_3_) was measured using an AutoMate FX automatic acidification preparation system attached to a CM-5012 CO_2_ coulometer in the Department of Oceanography at Texas A&M University. Approximately 15 mg of dried sample was placed inside glass tubes. The samples were automatically wetted with water and 10% H_3_PO_4_ and the resultant gas was delivered for measurement to the CM-5012 CO_2_ coulometer. ^230^Th-normalized carbonate fluxes were calculated by multiplying % calcium carbonate by the downcore ^230^Th-normalized MARs for 17JC from Loveley et al.^11^. An in-house sediment standard (‘Midway’) was included with each rack of sediments to check accuracy. Precision of the unknowns was estimated by repeating the analysis of every fifth sample in the sample run. One hundred one replicate analyses of % CaCO_3_ were used to calculate average reproducibility for the ^230^Th-normalized carbonate fluxes over the entire core. In doing so, we calculate a relative standard error of the carbonate flux replicate analyses of ± 0.09%.

Nannofossil carbonate was estimated as the fine (< 63 µm) fraction of carbonate^61–65^ in 17JC sediments. % nannofossil carbonate was calculated by subtracting the % coarse (> 63 µm) material from the % bulk carbonate for each sample. ^230^Th normalized nannofossil carbonate fluxes were calculated by multiplying the % nannofossil carbonate by the downcore ^230^Th-normalized MARS for 17JC from Loveley et al.^11^. The lack of foraminiferal fragmentation and likelihood of preferential preservation of carbonate in 17JC sediments (see Supplemental Sect. 1) at 17JC gives us confidence that the fine carbonate fraction of the sediments is majority nannofossils with little contribution from foraminiferal fragments. This is further corroborated by representative grain size analyses for the Holocene and LGM at 17JC^18^, that have shown that > 90% of the < 63 µm size fraction is found in the < 20 um fraction, which is most likely to be coccoliths^66–68^.

### Opal-carbonate ratios

Opal: (Opal + Nannofossil Carbonate) ratios were calculated by dividing the ^230^Th-normalized expected opal flux (see opal flux methods above) by the sum of the ^230^Th-normalized expected opal flux and ^230^Th-normalized nannofossil carbonate flux for each sample.

### xsBa and ^232^Th fluxes

^230^Th-normalized xsBa and ^232^Th fluxes from Loveley et al.^11^ were updated to reflect changes to the downcore ^230^Th MARs that resulted from updating the age model for core 17JC in this study.

## Supplementary Information

Supplementary Information 1.

Supplementary Information 2.

Supplementary Information 3.

Supplementary Information 4.

## Data Availability

Radiocarbon, [^231^Pa/^230^Th]xs, opal flux, and carbonate flux data will be archived at the National Oceanic and Atmospheric Administration National Centers for Environmental Information (NCEI) database upon publication and are also available as a supplement to this manuscript.
