# Supplementary material for: Insights into the deglacial variability of phytoplankton community structure in the eastern equatorial Pacific Ocean using [231Pa/230Th]xs and opal-carbonate fluxes
Source: Sci Rep. 2022 Dec 23;12:22258. doi: 10.1038/s41598-022-26593-1 (PMC9789155; doi:10.1038/s41598-022-26593-1)
Supplement: Supplementary file 3 — Supplementary Information 3. [file 41598_2022_26593_MOESM3_ESM.docx]

**Insights into the deglacial variability of phytoplankton community structure in the eastern equatorial Pacific Ocean using [^231^Pa/^230^Th]xs and opal-carbonate fluxes**

Danielle Schimmenti^1^, Franco Marcantonio^1^, Christopher T. Hayes^2^, Jennifer Hertzberg^3^, Matthew Schmidt^4^, John Sarao^1^

^1^ Department of Geology & Geophysics, Texas A&M University, College Station, TX USA

^2^ School of Ocean Science and Engineering, University of Southern Mississippi, Stennis Space Center, MS USA

^3^International Ocean Discovery Program, College Station, TX USA

^4^Department of Ocean and Earth Sciences, Old Dominion University, Norfolk, VA USA

**Supplementary Information**

1. **Calcite Preservation at 17JC:** The modern day lysocline occurs just below ~2900 m in the Panama Basin^1^, and there is evidence that it did not shoal above 2900 m in this region since the mid-Brunhes Transition ~430 ka^2^. At 2,846 m water depth, 17JC is right at or slightly above the lysocline where we would expect to observe an increase in the dissolution of calcite, affecting its preservation in the sediments. However, Mekik and Anderson (2018)^3^ have shown that calcite dissolution in EEP sediments is controlled by factors independent of bottom water CO_3_^2-^ saturation state and rain ratio (ratio of organic carbon flux to calcite flux). They find statistically significant inverse relationships between core top calcite dissolution and mass accumulation rates and between dissolution and focusing factors (this being the strongest) in the EEP such that sites with high sedimentation rates and focusing factors (ψ) are characterized by high calcite preservation and young core top ages. These authors suggest that syn-depositional lateral sediment focusing may help to preserve carbonate in sediments by increasing the supply of calcite to the sediments and therefore decreasing the fraction of carbonate that is dissolved. The youngest core tops and highest sediment focusing factors in the EEP are found in the Panama Basin. 17JC, specifically, is a focused site (average ψ = 4.1) with modern core top age (0 to 1 ka)^4^ and an average sedimentation rate of 17.5 cm kyr^-1^ for the past 25 kyr. We therefore estimate, based on interpolation following the relationship between MFI-based % calcite dissolved and ψ described in Mekik and Anderson (2018), as much as ~45% calcite dissolved in the sediments on average for the entirety of the core record (~83 kyr) and << 30% dissolved during the early Holocene and LGM when ψ reaches values upwards of 12 at 17JC.

Visual inspection of foraminifera tests in 17JC across the last 25 ka indicates good to excellent preservation of carbonates. Tests show no signs of dissolution pits or disintegration of chamber walls. Average shell weights of *G. ruber* from the 250-350 um size fraction are also very consistent (≤ 10% variation) across the LGM to Holocene (Supplemental Figure 1). Shell weights are, in fact, slightly higher during the LGM through deglacial compared to the Holocene indicating enhanced carbonate preservation and minimal fragmentation^5^ when nannofossil carbonate fluxes are highest at 17JC (see Figure 2; main text). As such, 17JC represents a unique locality where we can confidently employ carbonate fluxes as a proxy to reconstruct paleoclimatic/paleoceanographic change.

**Supplemental Figures & Tables**


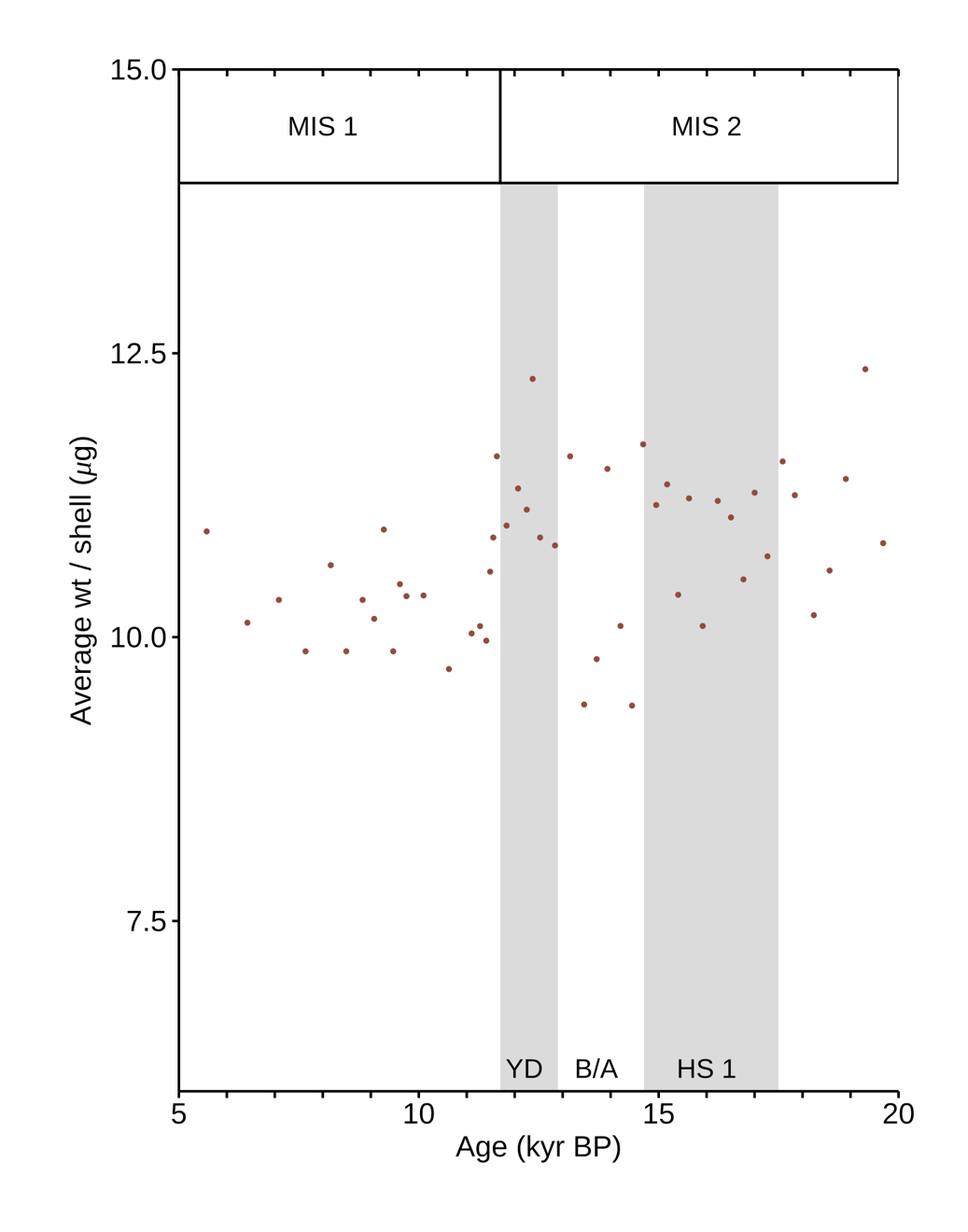


**Supplemental Figure 1.** Average shell weight per individual for *G. ruber* over the past 20 kyr at 17JC.

**Supplemental Table 1.** Core Locations, Depths, and Data Sources for data used in Supplemental Figure 2.

| **Core Site** | **Latitude** | **Longitude** | **Water Depth (m)** | **Data Sources** |
| --- | --- | --- | --- | --- |
| MV1014-02-17JC | 0.180495° S | 85.866737° W | 2846 | *This Study* |
| V19-30 | 3.383° S | 83.517° W | 3091 | *Dubois et al. (2010)*^6^*; Bradtmiller et al. (2006)*^7^ |
| ME0005A-27JC | 1.853333° S | 82.786667° W | 2203 | *Dubois et al. (2010)* |
| RC11-238 | 1.517° S | 85.817° W | 2573 | *Bradtmiller et al. (2006)* |
| RC13-140 | 2.867° N | 87.75° W | 2246 | *Bradtmiller et al. (2006)* |
| TR163-31P | 3.618667° S | 83.970667° W | 3025 | *Dubois et al. (2010)* |
| ME0005A-24JC | 0.021667° N | 86.463333° W | 2941 | *Dubois et al. (2010)* |
| RC13-114 | 1.65° S | 103.63° W | 3436 | *Bradtmiller et al. (2006)* |
| TR163-19P | 2.258333° N | 90.951667° W | 2348 | *Dubois et al. (2010)* |
| TR163-22P | 0.515° N | 92.398333° W | 2830 | *Dubois et al. (2010)* |
| V21-40 | 5.517° S | 106.767° W | 3182 | *Bradtmiller et al. (2006)* |

**
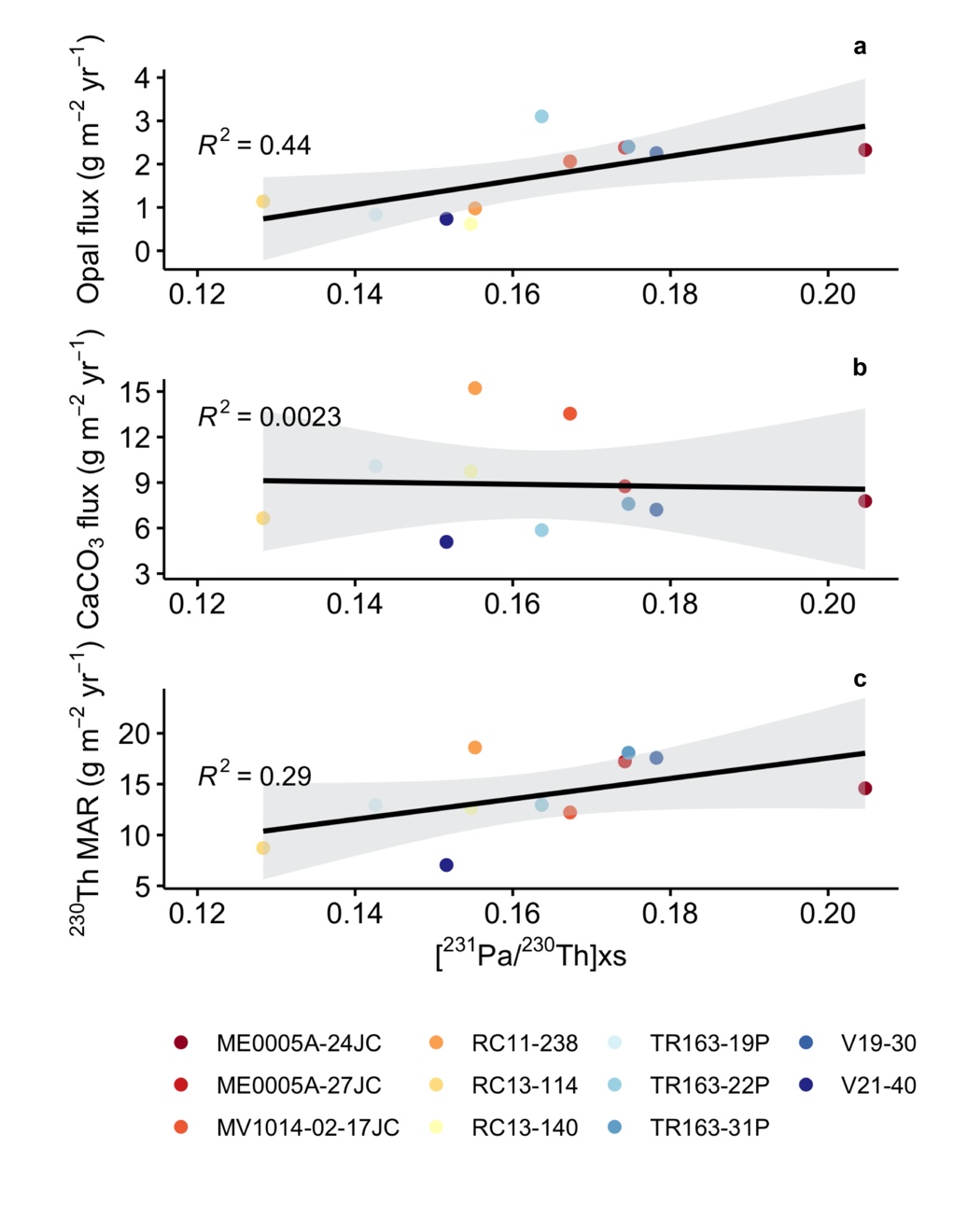
**

**Supplemental Figure 2.** Linear Regressions of Average Sedimentary Mass Accumulation Rates (MARs) or fluxes and [^231^Pa/^230^Th]xs ratios across the EEP for the last 25,000 years. **a)** Average [^231^Pa/^230^Th]xs ratios vs. Average Opal Fluxes (g m^-2^ yr^-1^) for each core site (R^2^ = 0.44) **b)** Average [^231^Pa/^230^Th]xs ratios vs. Average CaCO_3_ fluxes (g m^-2^ yr^-1^) for each core site (R^2^ = 0.0023) **c)** Average [^231^Pa/^230^Th]xs ratios vs. Average ^230^Th MARs (g m^-2^ yr^-1^) for each core site (R^2^ = 0.29). Grey shading around the regression line represents the 95% confidence interval for each regression. See Supplemental Table 1 for core locations and data sources.


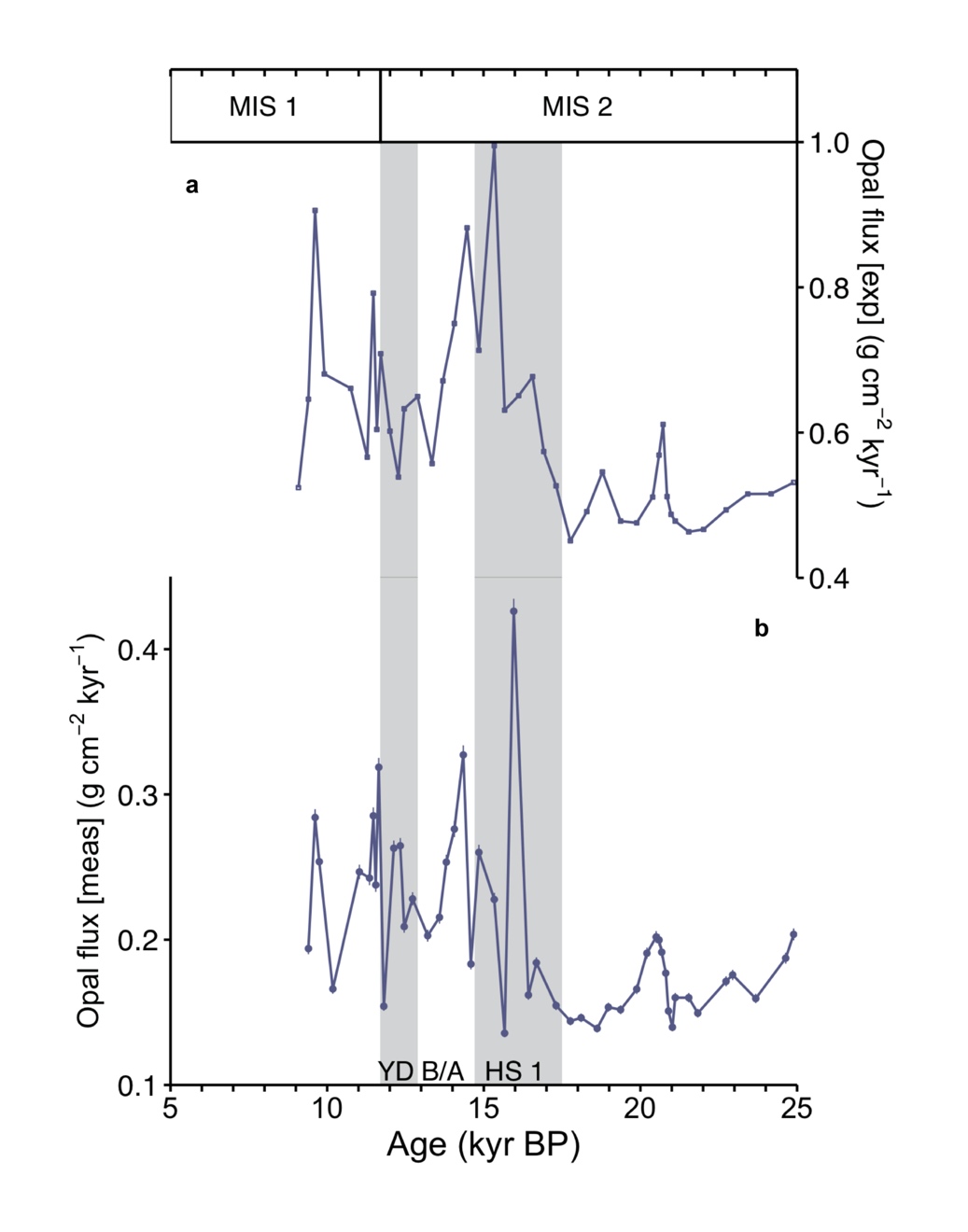


**Supplemental Figure 3.** 17JC Expected vs. Measured Opal Fluxes. **a)** Expected ^230^Th-normalized opal fluxes estimated using a detrital ^232^Th concentration of 14 ppm (see methods). **b)** Measured ^230^Th-normalized opal fluxes. MIS boundaries are from Lisiecki and Raymo (2005)^8^ and HS 1, B/A, and YD boundaries are from McManus et al. (2004)^9^ (grey shaded bars represent cold stadials).

**References**

1. Thunell, R. C., Keir, R. S. & Honjo, S. Calcite Dissolution: An in situ Study in the Panama Basin. *Science* **212**, 659–661 (1981).

2. López-Otálvaro, G.-E., Flores, J.-A., Sierro, F. J. & Cacho, I. Variations in coccolithophorid production in the Eastern Equatorial Pacific at ODP Site 1240 over the last seven glacial–interglacial cycles. *Marine Micropaleontology* **69**, 52–69 (2008).

3. Mekik, F. & Anderson, R. Is the core top modern? Observations from the eastern equatorial Pacific. *Quaternary Science Reviews* **186**, 156–168 (2018).

4. Marcantonio, F., Lyle, M. & Ibrahim, R. Particle sorting during sediment redistribution processes and the effect on 230Th-normalized mass accumulation rates. *Geophysical Research Letters* **41**, 5547–5554 (2014).

5. Hertzberg, J. E. & Schmidt, M. W. Refining Globigerinoides ruber Mg/Ca paleothermometry in the Atlantic Ocean. *Earth and Planetary Science Letters* **383**, 123–133 (2013).

6. Dubois, N. *et al.* Sedimentary opal records in the eastern equatorial Pacific: It is not all about leakage. *Global Biogeochemical Cycles* **24**, (2010).

7. Bradtmiller, L. I., Anderson, R. F., Fleisher, M. Q. & Burckle, L. H. Diatom productivity in the equatorial Pacific Ocean from the last glacial period to the present: A test of the silicic acid leakage hypothesis. *Paleoceanography* **21**, (2006).

8. Lisiecki, L. E. & Raymo, M. E. *Pliocene-Pleistocene stack of globally distributed benthic stable oxygen isotope records*. (PANGAEA, 2005). doi:10.1594/PANGAEA.704257.

9. McManus, J. F., Francois, R., Gherardi, J.-M., Keigwin, L. D. & Brown-Leger, S. Collapse and rapid resumption of Atlantic meridional circulation linked to deglacial climate changes. *nature* **428**, 834–837 (2004).
