# Supplementary material for: Insights into the deglacial variability of phytoplankton community structure in the eastern equatorial Pacific Ocean using [231Pa/230Th]xs and opal-carbonate fluxes
Source: Sci Rep. 2022 Dec 23;12:22258. doi: 10.1038/s41598-022-26593-1 (PMC9789155; doi:10.1038/s41598-022-26593-1)
Supplement: Supplementary file 4 — Supplementary Information 4. [file 41598_2022_26593_MOESM4_ESM.docx]

**Insights into the deglacial variability of phytoplankton community structure in the eastern equatorial Pacific Ocean using [^231^Pa/^230^Th]xs and opal-carbonate fluxes**

Danielle Schimmenti^1^, Franco Marcantonio^1^, Christopher T. Hayes^2^, Jennifer Hertzberg^3^, Matthew Schmidt^4^, John Sarao^1^

^1^ Department of Geology & Geophysics, Texas A&M University, College Station, TX USA

^2^ School of Ocean Science and Engineering, University of Southern Mississippi, Stennis Space Center, MS USA

^3^International Ocean Discovery Program, College Station, TX USA

^4^Department of Ocean and Earth Sciences, Old Dominion University, Norfolk, VA USA

**Supplementary Table Legends**

**Supplementary Data Table 2.** 17JC sample depths, ages, [^231^Pa/^230Th^]xs ratios, ^230^Th-normalized opal, bulk carbonate, and nannofossil carbonate fluxes

**Supplementary Data Table 3.** 17JC radiocarbon data
